# Foldable and Disposable Memory on Paper

**DOI:** 10.1038/srep38389

**Published:** 2016-12-06

**Authors:** Byung-Hyun Lee, Dong-Il Lee, Hagyoul Bae, Hyejeong Seong, Seung-Bae Jeon, Myung-Lok Seol, Jin-Woo Han, M. Meyyappan, Sung-Gap Im, Yang-Kyu Choi

**Affiliations:** 1 School of Electrical Engineering, Korea Advanced Institute of Science and Technology, (KAIST) 291 Daehak-ro, Yuseong-gu, Daejeon 34141, South Korea; 2 Department of Memory Business, Samsung Electronics, San #16 Banwol-Dong, Hwasung-City, Gyeonggi-Do 445-701, Republic of Korea; 3 Department of Chemical and Biomolecular Engineering, Korea Advanced Institute of Science and Technology (KAIST), 291 Daehak-ro, Yuseong-gu, Daejeon 305-701, Republic of Korea; 4 Graphene Research Center, KI for Nanocentury, KAIST, Daejeon 34141, South Korea; 5 Center for Nanotechnology, NASA Ames Research Center, Moffett Field, CA 94035, USA

## Abstract

Foldable organic memory on cellulose nanofibril paper with bendable and rollable characteristics is demonstrated by employing initiated chemical vapor deposition (iCVD) for polymerization of the resistive switching layer and inkjet printing of the electrode, where iCVD based on all-dry and room temperature process is very suitable for paper electronics. This memory exhibits a low operation voltage of 1.5 V enabling battery operation compared to previous reports and wide memory window. The memory performance is maintained after folding tests, showing high endurance. Furthermore, the quick and complete disposable nature demonstrated here is attractive for security applications. This work provides an effective platform for green, foldable and disposable electronics based on low cost and versatile materials.

Continued advancement of information technology (IT) in the future demands novel electronic devices that provide versatile functionality beyond mere performance enhancement driven by silicon-based complementary metal-oxide-semiconductor (CMOS) technology. In this regard, flexible electronics has recently garnered considerable attention due to attractive features and potential for versatile applications such as transistors^1^,^2^,^3^, actuators^4^,^5^,^6^,^7^, displays^8^,^9^,^10^, and sensors^11^,^12^. Such flexible devices have also become the foundation for wearable electronics^13^. A substantial amount of research has been devoted to showing the feasibility to fabricate flexible devices^14^,^15^,^16^, where a flexible substrate plays an important role as a fundamental component. Various kinds of polymers such as polymide (PI), poly ethylenenaphthalate (PEN), and poly ethyleneteraphthalate (PET) have been used to date as the substrate^17^,^18^,^19^. However, plastics accompany environmental issues owing to poor biodegradability and additional cost for disposal after use^20^. It is therefore essential to find an eco-friendly and biodegradable substrate. In contrast to plastics, paper easily decays without giving rise to environmental concerns and has therefore been recently exploited as a useful substrate for electronic devices^20^,^21^,^22^, resulting in paper electronics. It has several advantages compared to silicon and plastic substrates, with low raw material cost being the foremost. Furthermore, the fabrication cost and time to make a device can also be reduced with the aid of modern high throughput printing techniques such as roll-to-roll processing. Moreover, paper is easily foldable and thus has become a potential substrate for flexible electronics that provides not only bendability but also foldability.

In the midst of a great variety of electronic components being implemented on paper, memory devices are not an exception. Memory device is a fundamental component of most electronic systems, enabling information storage via a writing-holding-erasing process and a pre-setting apparatus to enable a control unit^23^,^24^. Commercial implementation of paper-based memory that is capable of repetitive programming and erasing operation faces a few issues. First, it is difficult to supply continuous power to the memory fabricated on paper. Also, complicated and harsh fabrication processes typical with semiconductor substrates should be avoided. Therefore, non-volatility without a constant power supply and structural simplicity based on simple processing is needed for paper-based memory. Among the various types of non-volatile memory, resistive random access memory (RRAM) is considered as a next generation non-volatile memory due to its fast access speed, high density and easy fabrication process^25^,^26^. In particular, a metal-insulator-metal (MIM) structure such as capacitor, can be made by simple fabrication processes compared to other classes of non-volatile memory. In addition to non-volatility and a simple structure, a wide choice of materials is also important with regard to flexibility. Organic materials with superior elasticity and mechanical bendability and attributes of low-cost and low-temperature fabrication have already contributed to the growth of flexible electronics^26^,^27^,^28^. In this regard, a hybrid of RRAM and organic electronics is attractive for paper-based memory which is considered here.

The fabrication of organic memory on ordinary paper, however, is very challenging. Above all, the rough and porous surface of the paper is an inherent obstacle for fabricating microscale devices^29^. Ordinary paper also cannot accommodate a conventional silicon process based on high temperature, wet cleaning and intense chemical treatment. In particular, paper is vulnerable to typical solution process-oriented polymerization where such processes are used when an organic layer, serving as the resistive switching medium, is formed. Meanwhile, there are alternative routes to realize paper-based memory. For example, the entire device can be fabricated on a silicon substrate and then transferred to a paper substrate. However, additional cost and time are involved in such transfer approaches. Therefore, employing an appropriate type of paper and developing a process compatible with paper are the most crucial considerations for successful paper electronics.

In this study, we demonstrate foldable organic memory built on a nano-fibrillated cellulose paper (abbreviated as nanopaper). The memory fabricated on nanopaper can encompass a large variety of functional electronics with directly printable, disposable and foldable characteristics. Disposability stemming from the ability of the nanopaper to rapidly decay is advantageous to avoid environmental pollution and attractive for security systems against hacking because it affords complete destruction of the memory. The nanopaper has a root mean square (RMS) surface roughness on the nanometer scale and high foldability^30^,^31^,^32^,^33^. An organic layer, serving as the resistive switching layer (RSL), is deposited by initiated chemical vapor deposition (iCVD), which is an all-dry vapor-phase process that does not require any solvent^34^. Fabrication processes involving solvents lead to safety and health considerations, which can limit biocompatible applications. However, iCVD is a versatile process enabling a solvent-induced damage free, pure, well-adhered, conformal, ultra-thin (<10 nm) coating^35^,^36^,^37^. Furthermore, an all-dry polymerization process at room temperature allows thin film deposition on nearly all substrates, compared to previous reports depending on solution processing^38^,^39^,^40^,^41^. iCVD-grown polymer-based functional electronic devices have already been reported^42^. The all dry approach used here is a key advantage for ease of manufacturing based on paper electronics.

Paper based memory devices with foldable characteristics have been previously reported in the literature^31^,^38^,^40^,^43^. Antennas were fabricated on various types of papers and their conductivity degradation owing to folding was evaluated^31^. But, that study focused only on conductive metal line using silver nanowires and did not conduct cyclic folding and unfolding endurance test. In contrast, here we demonstrate cyclic endurance beyond 10^2^ times using fully functional memory devices. Another study in ref. 40 demonstrated cyclic foldability of an organic field effect transistor based non-volatile memory fabricated on a polyimide substrate. Despite its immunity against folding and bending stresses, the polyimide substrate is known as thermally stable material so that it is not appropriate for flame burning or water melting transient electronics. The nanocellulose paper in the present work not only enables foldability but also vanishes under fire. Bendability but not foldability of RRAM was demonstrated with the device fabricated on aluminum foil as a substrate and the cellulose nanofiber paper as the resistive switching material^43^. The ductility of the aluminum substrate makes it inappropriate for cyclic harsh folding applications. In addition, forming voltage greater than 10 V was necessary for a 50 μm × 50 μm device because the thickness control of the nanocellulose paper was presumably difficult and thus the device used a micron-thick resistive switching film. However, the iCVD method here allows precise control of the film and consequently all device functions are demonstrated with operational voltages below 1.5 V, which is adequate with battery operation. Lien *et al*. demonstrated a resistive switching memory printed on commercial copy paper with non-flammable inorganic material, i.e., titanium oxide as the RSL^38^. Disposability was demonstrated by burning the device but the residues still remained. The burnable nanocellulose substrate with the flammable organic RSL here provides comparable performance to that in ref. 38, however, it allows complete vanishing of the device upon burning. Moreover, our device permits lower voltage operation relative to titanium dioxide and retains the advantages of an all dry vapor phase process even in a use of organic resistive switching materials, which is due to iCVD technique.

Here, we used poly (ethylene glycol dimethacrylate) (pEGDMA) based iCVD for the RSL layer formation. The RSL polymer has a highly cross-linked structure^44^ with a proper band gap and a dielectric constant similar to that of silicon dioxide (refer to Supplementary Information S1). Top and bottom electrodes were patterned using direct inkjet printing without any complex photolithography or etching process. This process is crucial for paper electronics application due to its low cost and facile and rapid implementation^29^. Based on the aforementioned reliable processes and materials, RRAM was fabricated on the nanopaper. Its fundamental performance metrics including program/erase/read operations and endurance against ordinary bending cycles were assessed along with the foldability and disposability characteristics. In addition, the switching mechanism of the fabricated device corresponds to the filamentary conduction, where details are provided in Supplementary Information (refer to Supplementary Information S3)^34^.

## Results and Discussion

**Figure 1:**
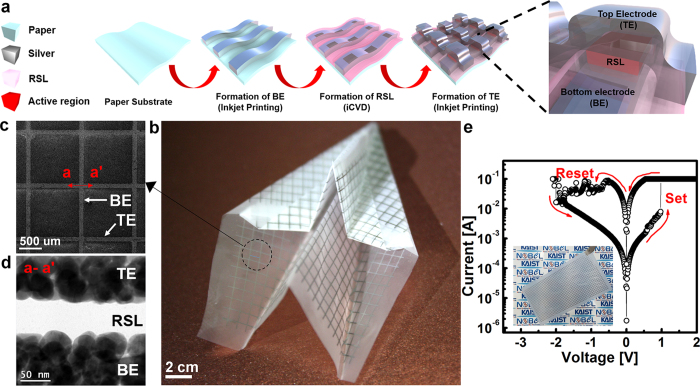
Fabrication process of paper substrate-based memory. (**a**) Schematics of the fabrication process. Inkjet printing and room temperature iCVD are suitable for fabricating the device on a paper substrate. (**b**) Fabricated nanopaper-based memory. The memory is displayed in the form of an airplane prepared by folding, i.e., origami, demonstrating the foldable memory feature. (**c**) Top view SEM image of the fabricated device. (**d**) Cross-sectional TEM image of the direction along a-a′ of Fig. 1(c). (**e**) I-V characteristic of the fabricated device showing memory operation. The inset image shows the nanopaper based memory before origami.

The simple fabrication procedure of the paper-based organic RRAM is illustrated in Fig. 1(a). Three different papers, i.e., the nanopaper as the preferred material and a polymer-coated glossy paper^20^ (i.e. photo paper) and an adhesive label^38^ (i.e. sticker) as control groups, were used here. Most descriptions below are for the nanopaper unless otherwise specifically stated. A bottom electrode (BE) made of silver (Ag) was directly patterned on the nanopaper using an inkjet printing technique, followed by RSL deposition using iCVD on the BE. Finally, the same inkjet-printed top electrode (TE) made of Ag was formed in perpendicular to the BE to create a crossbar array. The nanopaper-based foldable memory, which is in the form of an airplane by means of origami, is shown in Fig. 1(b). Figure 1(c) shows a top-view scanning electron microscopy (SEM) image at the cross point of the TE and the BE. The cross-sectional transmission electron microscopy (TEM) image (Fig. 1(d)) clearly shows a 50 nm-thick iCVD-based organic RSL. The uniformity of the RSL confirms the superior coating capability of the iCVD. The Ag electrode is well formed on both sides of the RSL. The components of the memory, the Ag electrodes and carbon based RSL, are confirmed by energy dispersive X-ray spectroscopy (EDS) mapping (refer to Supplementary Information S2). The representative memory characteristics are shown in Fig. 1(e) along with an image of the nanopaper-based memory prior to folding the origami. The memory is at an off-state with a high resistance state (HRS) before SET. With an abrupt increase of current after SET, it is at an on-state with a low resistance state (LRS). The memory is then returned to the HRS, i.e., the off-state by applying a reverse bias, called RESET. The memory exhibits a low operation voltage near ±1 V and an on-off current ratio of 10^2^, which represents the memory window.

**Figure 2:**
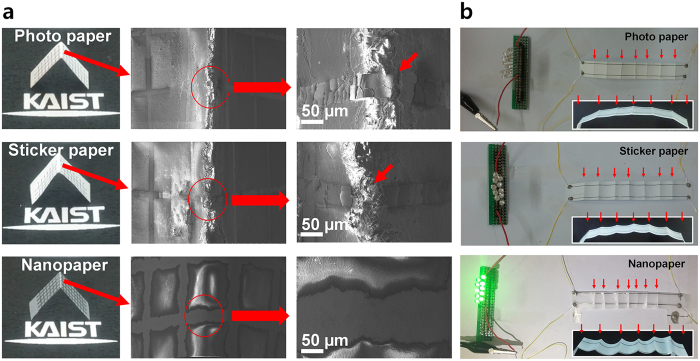
Folding test using various paper substrates. (**a**) SEM images of folded photo paper, sticker paper, and nanopaper. The arrows in the enlarged images indicate obvious ruptures of the Ag line folded on photo paper and sticker paper, respectively. (**b**) Verification of foldability via an electrical experiment. Under the folded state of the Ag inkjet-printed line, only the LED bulbs connected to the line formed on nanopaper sustained an ON state, demonstrating excellent foldability of the nanopaper.

Compared to a plastic substrate, another strength of the paper substrate is its foldability which is important in portable applications^45^,^46^. However, in contrast to flexible and rollable electronics realized on smoothly curved surface^47^,^48^, maintaining stable electrical characteristics and mechanical continuity of an initial pattern under a folded state is challenging. A susceptible region for failure is at the folded plane. When a folding event takes place, the dimension relevant to the folding plane would experience deformation on the order of microns. Therefore, the microscopic fragility and brittleness of the paper substrate are important factors to determine the robustness against folding. Paper with minimal roughness is therefore crucial for foldable electronics. In this regard, the nanopaper is a promising substrate for future foldable electronics. Figure 2 shows an Ag line pattern on three different paper substrates and their electrical stability upon folding. Figure 2(a) shows SEM images taken at the top of the folding plane. When the paper was folded, the Ag lines formed on the photo paper and the sticker paper snapped owing to a crack on the paper surface. In these cases, the two papers were coated with polymer on the surface and it has been shown previously that such polymer coating leads to the loss of random network structure which in turn results in cracks formed at the surface^49^. Such randomly oriented network structure leads to high foldability, resulting in a decrease of damage^50^. Nanopaper on the other hand, without such polymer coating, is able to preserve the initial patterned state without the loss of electrical stability even under the folded condition. The high tolerance of the nanopaper against folding is directly verified by an electrical experiment using LED bulbs (Fig. 2(b)). Unlike the photo paper and the sticker paper, the LED bulb connected to a power supply via the Ag line formed on the nanopaper was successfully turned on after iterative folding exceeding hundred times. This result confirms that nano-particle-based inkjet printing can be successfully performed on nanopaper without a polymer coating to improve the surface roughness.

**Figure 3:**
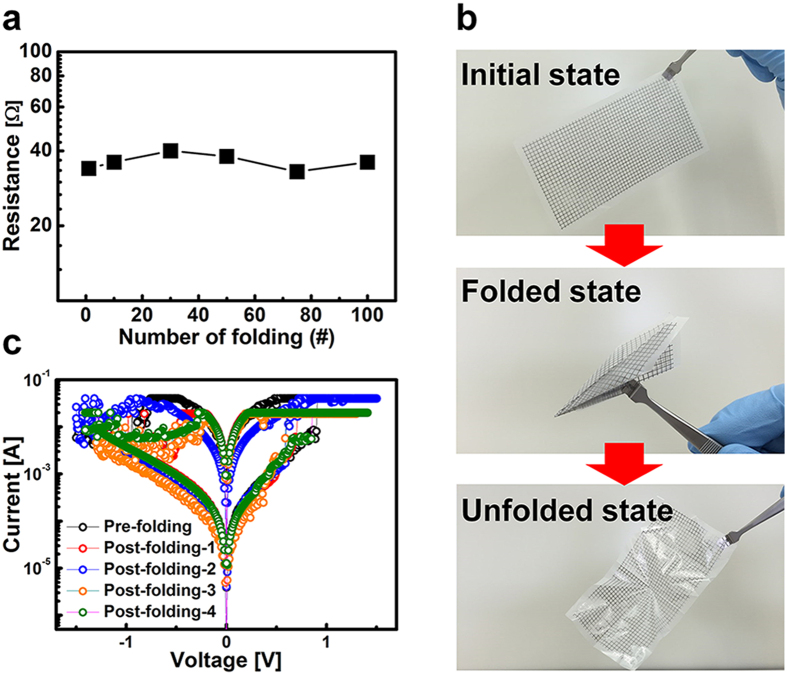
Folding endurance and origami experiment to prove foldable memory. (**a**) Resistance of Ag inkjet-printed line as a function of number of folding iterations. (**b**) Origami using nanopaper-based memory. (**c**) I-V characteristics of nanopaper-based memory before and after origami experiment, showing no significant degradation of the memory operation after origami.

Figure 3(a) shows the folding reliability of the RRAM on the nanopaper with inkjet-printed patterns. It exhibits no noticeable change in resistance of the Ag line formed on the nanopaper with up to 10^2^ folds. A paper airplane was made by origami in order to demonstrate the extent of the foldable nature of the memory fabricated on the nanopaper. Figure 3(b) shows an image of the memory before and after making the origami. Finally, the memory function was evaluated in the unfolded state again. As shown in Fig. 3(c), the device exhibits stable memory operation regardless of folding. It should be noted that the measured cell was running parallel along the folded line. The unfolded memory after making the origami shows nonvolatile and reliable characteristics up to 10^2^ switching cycles, indicating an acceptable memory window (ratio of high to low resistance states) of 10^2^ and a uniform operation voltage distribution near 1 V (refer to Supplementary Information S3). The present memory fabricated on nanopaper not only demonstrates the feasibility of foldable electronics but is also expected to lead to the realization of array-based high-capacity memory with foldability.

**Figure 4:**
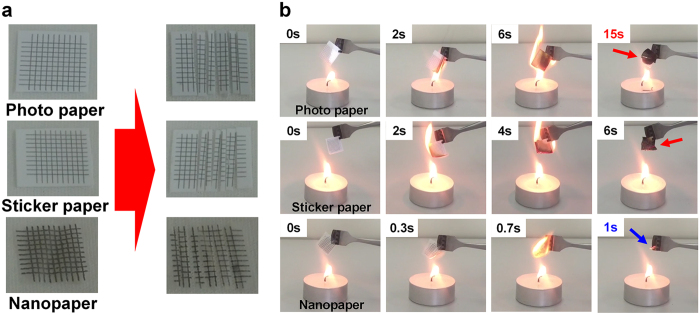
Testing of disposable memory. (**a**) Images of each memory with various paper substrates before and after severing. The paper substrate-based devices can be easily severed using a knife and scissors. (**b**) Physical destruction of the memory using incineration. The nanopaper-based memory showed the clearest destruction in the shortest burning time.

Physical destruction of the memory is the safest method for information security against hacking. However, discarding conventional memory involves a complicated process that is time-consuming and costly due to the sturdy semiconductor-based substrate. In this regard, a paper substrate is suitable for easy disposability of the electronic device. Figure 4 shows this feature via a simple process. The memory fabricated on various papers - photo paper, sticker paper, and nanopaper - was easily destroyed by severing with a scissor, knife, or a shredder (Fig. 4(a)). For disposable electronics, vulnerability to fire can be favorably exploited for permanent destruction of documents. Figure 4(b) shows the simple disposal process using incineration, where the memory on all paper substrates was completely burned in a few seconds, showing easy and fast disposability. In particular, the RRAM on the nanopaper was burned most rapidly within 1 second (refer to Supplementary Information S4). This results from the low thickness and inherent material property of the wood-based cellulose product without a coated polymer. Short disposable time is an important figure of merit for disposable electronics aiming at hardware based security applications. When considering environmental problems, destruction of the nanopaper-based memory using incineration is another useful benefit. As there is no detrimental plastic coating on the wood-based nanopaper, unlike the other two, the nanopaper substrate is attractive for disposable green electronics.

**Figure 5:**
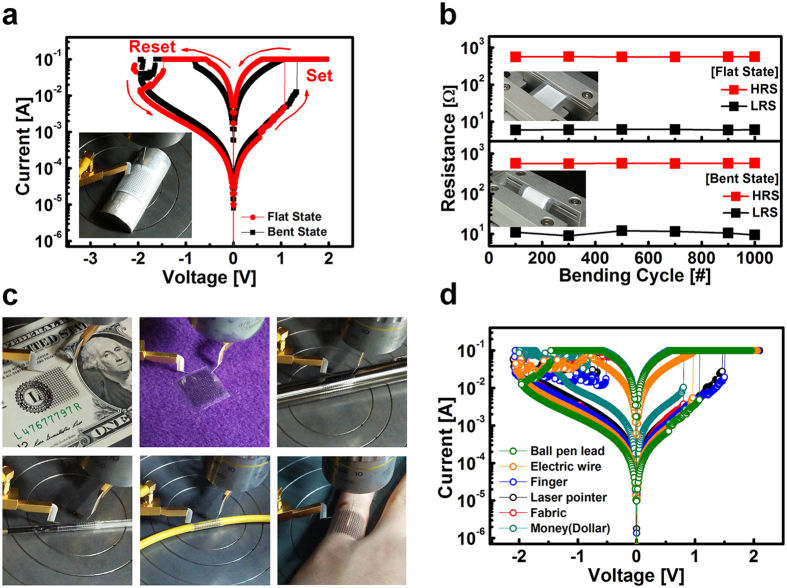
Results of bendability and application to sticker memory. (**a**) I-V characteristics before and after bending, in which a custom-made bending mold was used as shown in the inset image. (**b**) The variation of HRS and LRS, i.e., the memory window, as a function of bending cycles. (**c**) Easily attachable sticker memory on each structure with various curvatures. The sticker memory was fabricated on a transparent adhesive label. (**d**) I-V characteristics of the sticker memory. The sticker memory exhibited stable memory operation regardless of the various curvatures of the used structures.

A home-made hemi-cylinder with a radius of 10 mm was used to evaluate the memory operations under a bent state. As shown in Fig. 5(a), the memory fabricated on the photo paper exhibits stable memory operation under the bent state. Figure 5(b) shows distinctive resistance at HRS and LRS, i.e., the memory window, as a function of the bending cycles. The two curves indicate the variation of resistances under the flat state and bent state, respectively, after iterative bending cycles. The radius of curvature of the sample was 10 mm at the bent state. Bending cycles of more than a thousand did not cause degradation within the sensing window, which implies that the extrapolated endurance would allow flexible applications. In order to demonstrate a memory aimed at smart patch applications, the memory was fabricated on an adhesive label, i.e., a transparent sticker. The sticker memory was easily and tightly attached to various target substrates as shown in Figure 5(c), exhibiting stable memory operation (Fig. 5(d)). The sticker memory is a multipurpose device due to its good adhesion to various geometric surfaces, and the feature of organic memory supports more reliable bendability. For instance, memory affixed on a bank note can be helpful for monitoring cash flow and tracking the domestic economy. Memory on a fabric meanwhile can permit applications for wearable smart clothes. In particular, the sticker memory was also adhered to human finger, as shown in Fig. 5(c). The sticker memory on skin opens the possibility of epidermal electronics on the basis of the use of room temperature-based iCVD technique. The sticker memory shows extraordinary bendability on a ball pen lead with a radius of 1.5 mm, which illustrates extreme bendability. Figure 5(c) shows the versatility of the paper based RRAM for wearable smart device applications.

In summary, we have demonstrated nanopaper-based foldable organic nonvolatile memory with versatile functionality based on low cost, easy and fast process. The nanopaper consisting of pure nano-fibrillated cellulose shows nanoscale surface roughness and high foldability, which plays a crucial role in realizing foldable organic memory. Inkjet printing technology permits easy and fast patterning of electrodes without complex photolithography or etch processes. Employing a room temperature-based iCVD process, which can be used to cover virtually any substrate, enables demonstration of the first nonvolatile organic memory based on all-dry polymerization process, encompassing organic electronics and paper electronics. As a result, the inkjet-printed pattern formed on nanopaper shows stable conductivity under a folded state, and the nanopaper-based memory exhibits reliable nonvolatile memory performance even after the extent of folding the device into origami. The present demonstration provides a gateway for future developments toward fabricating flexible devices that can endure physical deformation beyond foldability. Furthermore, the nanopaper shows the most rapid disposability among all types of papers; this feature is attractive for disposable electronics aimed at security applications. In addition, organic memory on a sticker shows the potential of paper electronics for versatile smart applications. Thus, this study provides a platform for foldable electronics and ensuing applications embracing green, disposable, and wearable electronics.

## Methods

### Fabrication of the memory on various paper substrates

Various papers were used as substrates for device fabrication in this study: Photo paper (Advanced photo paper, HP), transparent sticker (V3960-5, Printec), sticker paper (V6510-10, Printec), and nanopaper (Nanocellulose films, VTT, Finland). Three papers excluding the nanopaper are commercially available. The surface morphology of all papers was analyzed by atomic force microscopy (AFM) (refer to Supplementary Information S5). Two electrodes were formed using a low cost inkjet printing technique that does not require a mask for photolithography. In order to form the top and bottom electrodes, Ag was deposited by an inkjet printer (UJ200MF, Unijet) integrated with a piezoelectric type head (MicroFab) and 50-μm-orifice nozzles. A conductive nanoparticle Ag ink (InkTec Tec-IJ-060) was used at room temperature in air. Prior to ink-jet printing, solutions were filtered through a 5 μm syringe filter to ensure that very large aggregates of Ag nanoparticles were not used in the ink-jet nozzles. As a result, the average particle size of the colloids was about 150 nm. Typical drop volumes were in the range of 30 to 40 pL. The diameter of the droplets was around 80 μm, which determined a drop pitch in the range of 70 to 90 μm. The pEGDMA used as the RSL was polymerized by an all-dry vaporization based iCVD technique under room temperature. Ethylene glycol dimethacrylate (EGDMA, 97%, Sigma Aldrich) and tert-butyl peroxide (TBPO, 97%, Sigma Aldrich) were employed as the monomer and the initiator, respectively. After vaporization of EGDMA and TBPO, both materials were delivered to a custom-made iCVD reactor. The process pressure was 70 mTorr and the filament was heated to 200 °C. The flow rate of the EGDMA and the TBPO was maintained at 1 sccm by a needle valve. The substrate temperature was maintained at nearly room temperature for the full process. The deposition rate of the pEGDMA layer was 5 nm/min. The characterization of pEGDMA using Fourier transform infrared spectroscopy (FT-IR) shows successful polymerization of the EGDMA monomer (refer to Supplementary Information S6).

### Analysis and characterization

In order to prepare the sample for TEM, a focused ion beam (FIB, model Helios Nanolab) was employed after coating carbon and platinum for passivation of the sample. The cross-sectional image was obtained from high-resolution TEM (model JEM-ARM200F), and the components of the memory were analyzed by EDS mapping (model Quantax 400). All electrical measurements were carried out without any device encapsulation. The I-V characteristics were measured using a HP4156 semiconductor parameter analyzer. A custom-made curved mold (in inset of Fig. 2(a)) and a bending machine (in inset of Fig. 2(b)) were used to evaluate the flexibility and the bending endurance, respectively. SEM (model S-4800) was employed for visual analysis of the folded structure fabricated on photo paper, sticker paper, and nanopaper substrates. In order to demonstrate stable foldability of an Ag line printed on nanopaper, a light-emitting diode (LED) bulb and a proper power supply were used, where Ag paste permitted good contact between the electric wire and the Ag line. The surface morphology of each paper was analyzed using AFM (model XE100). FT-IR analysis was employed to confirm successful polymerization of the EGDMA monomer. Electron energy loss spectroscopy (EELS, model: Gatan Enfina) was used to extract the energy band-gap of the pEGDMA.

## Additional Information

**How to cite this article**: Lee, B.-H. *et al*. Foldable and Disposable Memory on Paper. *Sci. Rep.* **6**, 38389; doi: 10.1038/srep38389 (2016).

**Publisher's note:** Springer Nature remains neutral with regard to jurisdictional claims in published maps and institutional affiliations.

## Supplementary Material

Supplementary Information
