# Supplementary material for: Foldable and Disposable Memory on Paper
Source: Sci Rep. 2016 Dec 6;6:38389. doi: 10.1038/srep38389 (PMC5138845; doi:10.1038/srep38389)
Supplement: Supplementary Information [file srep38389-s1.doc]

Supplementary Information for:

Foldable and Disposable Memory on Paper

*Byung-Hyun Lee, ^1, 2†^, Dong-Il Lee, ^1†^, Hagyoul Bae, ^1^ Hyejeong Seong, ^3, 4^ Seung-Bae Jeon, ^1^ Myung-Lok Seol, ^5^ Jin-Woo Han, ^5^ Meyya Meyyappan, ^5^ Sung-Gap Im, ^3,4^ and Yang-Kyu Choi^1^**

^1^School of Electrical Engineering, Korea Advanced Institute of Science and Technology, (KAIST) 291 Daehak-ro, Yuseong-gu, Daejeon, 34141, South Korea

^2^Department of Memory Business, Samsung Electronics, San #16 Banwol-Dong, Hwasung-City, Gyeonggi-Do 445-701, Republic of Korea

^3^Department of Chemical and Biomolecular Engineering, Korea Advanced Institute of Science and Technology (KAIST), 291 Daehak-ro, Yuseong-gu, Daejeon 305-701, Republic of Korea

^4^Graphene Research Center, KI for Nanocentury, KAIST, Daejeon, 34141, South Korea ^5^Center for Nanotechnology, NASA Ames Research Center, Moffett Field, CA 94035, USA

† These authors equally contributed to this work

*** Authors to whom correspondence should be addressed.

Email addresses: [ykchoi@ee.kaist.ac.kr](mailto:ykchoi@ee.kaist.ac.kr)

**Table of contents**

1. **Extraction of the energy band gap of pEGDMA.**
2. **EDS mapping to analyze the components of the device.**
3. **Memory effect after folding experiment using origami.**
4. **Experimental result for disposable memory.**
5. **Surface morphology of papers used as a substrate.**
6. **Characterization of pEGDMA.**

**1. Extraction of the energy band gap of pEGDMA**

The chemical structure of poly ethylene glycol dimethacrylate (pEGDMA) consists of carbon and oxygen, as illustrated in Figure S1(a). A 100-nm-thick pEGDMA film on 100-nm-thick silicon dioxide (SiO_2_) grown on a Si wafer was prepared to extract the energy band-gap (*E_G_*) of pEGDMA. A sample for SiO_2_ with the same thickness was prepared to verify the analysis result of pEGDMA. Electron energy loss spectroscopy (EELS) built into a Cs-corrected scanning transmission electron microscope (Cs-STEM, ARM 200) was employed to analyze the E_G_ of the samples. Figure S1(b) shows the energy band-gap (*E_G_*) of pEGDMA extracted from the EELS analysis. The E_G_ of SiO_2_ extracted by the same method is 8.73 eV, which is approximately equal to the reported value of 8.9 eV. The close verification for SiO_2_ supports the validity of our method to extract a reasonable *E_G_* for pEGDMA.


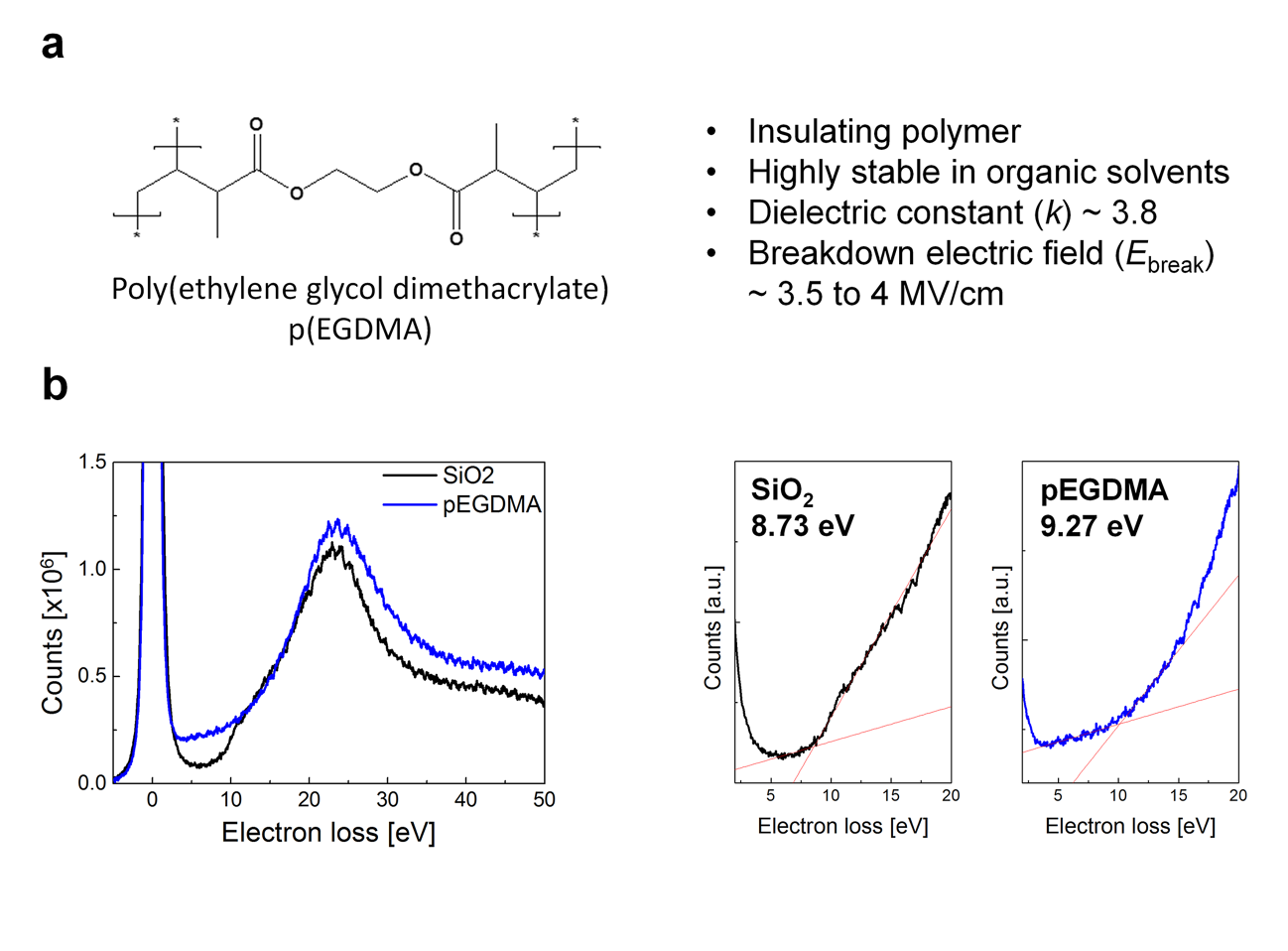


**Figure S1**. Chemical structure and E_G_ of pEGDMA. (a) Chemical structure of pEGDMA and basic features as a dielectric. (b) Energy band-gap of pEGDMA. The E_G_ value of SiO_2_ extracted by the same method is close to the reported value, which supports the ability to extract a reliable E_G_ value of pEGDMA

**2. EDS mapping to analyze the components of the device**

The fabricated device consists of a resistive switching layer (pEGDMA) sandwiched between two metal electrodes (silver), which is shown in Figure S2, obtained using energy dispersive spectrometer (EDS) mapping


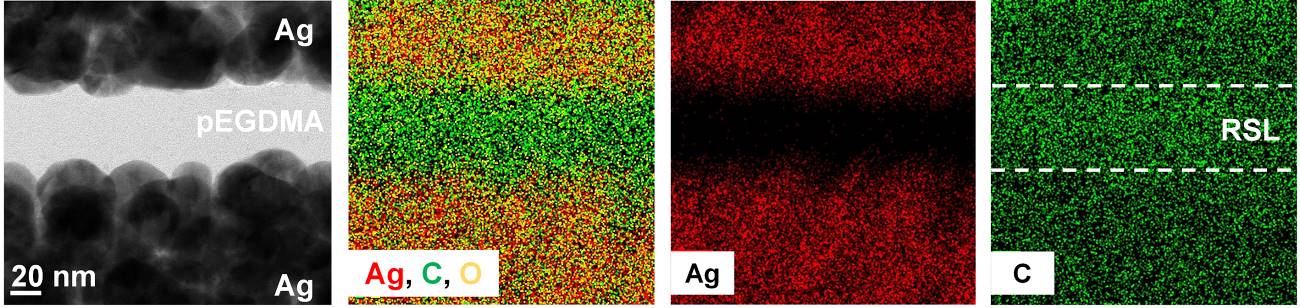


**Figure S2**. Analysis of each layers of the fabricated device using EDS mapping.

**3. Memory effect after origami folding experiment**

The nanopaper-based organic memory exhibited stable memory operation even after an origami folding experiment. In order to verify reliable application for foldable electronics, an additional electrical measurement was carried out. The nanopaper memory used in the origami experiment showed stable memory operation for hundred switching cycles without significant performance degradation (Figure S3(a)). Simultaneously, low operation voltage (1 V) with an acceptable voltage distribution was also confirmed (Figure S3(b)). As expected, nanopaper memory, which operates as resistive random access memory (RRAM), exhibited the feature of nonvolatile memory (Figure S3(c)). In order to analyze the memory switching mechanism, the *I-V* characteristics were replotted on a log-log scale, as shown in Figure S3(d). The switching mechanism of the memory corresponds to filamentary conduction, which is supported by the absence of dependency on cell size (Figure S3(e), S3(f)).^1^ More specifically, the switching mechanism of the organic RRAM with the same structure, which is the metal-insulator-metal structure with the pEGDMA resistive switching layer sandwiched between inkjet-printed silver electrodes, was carbon filamentary conduction, as reported in our previous work.^2^ Figure S3(g), S3(h), and S3(i) show a dispersion of main memory parameters such as switching endurance, retention time, and memory window, where memory window was the difference of the resistance between a high resistance state (HRS) and a low resistance state (LRS) to identify a memory state.

**Figure S3**. Memory effect of nanopaper memory after origami experiment. (a) Variation of resistance at HRS and LRS as a function of switching cycles. It shows the variation of the memory window for multiple switching operations. (b) Distribution of the operation voltage obtained from an endurance test. (c) Variation of the memory window as a function of time after the switching endurance test, which shows the nonvolatile nature of the nanopaper memory. (d) *I-V* characteristics with logarithmic type to interpret the switching mechanism of RRAM. (e) Dependency on cell size of nanopaper memory. (f) Variation of operation voltage as a function of cell size. As shown in Figure (e), (f), even though the cell size is changed, there is no significant difference in the memory operation. (g) Distribution of the switching endurance characteristic. (h) Distribution of the data retention characteristic. (i) Distribution of the HRS and LRS for identifying a memory state.

**4. Experimental evidence for disposable memory**

As shown in the embedded video file, the nanopaper memory boasts the fastest physical destruction among the memory devices on various paper substrates, making it suitable for disposable memory aimed at high security.


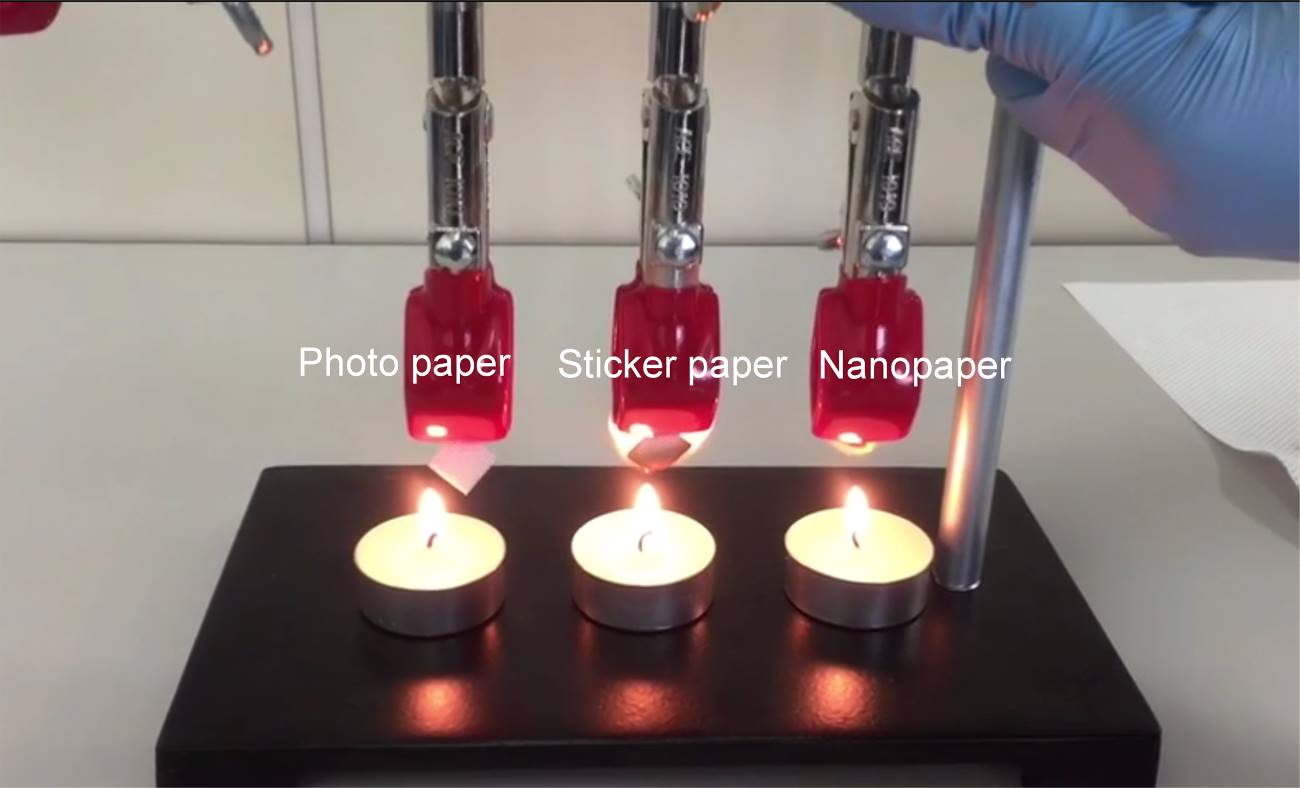


**Figure S4**. Snapshot during incineration test to evaluate the disposability of various memory devices.

**5. Surface morphology of papers used as substrate**

The paper as a substrate for electronic devices requires high flatness. The surface morphology of various papers was analyzed by atomic force microscopy (AMF, model: XE100). As expected, due to the polymer coated on pure cellulose paper, the photo paper and sticker paper show high flatness with nanoscale root mean square (RMS). Otherwise, pulp paper without a polymer coating has severe surface roughness at a microscale level (Figure S5(b)), resulting in failure of patterning using conductive ink consisting nano-particles.^3^ Even though it is only a wood-based paper without polymer coating, the nanopaper shows a smooth surface roughness with nanoscale RMS (Figure S5(b)), thereby permitting fabrication of nanoscale device.


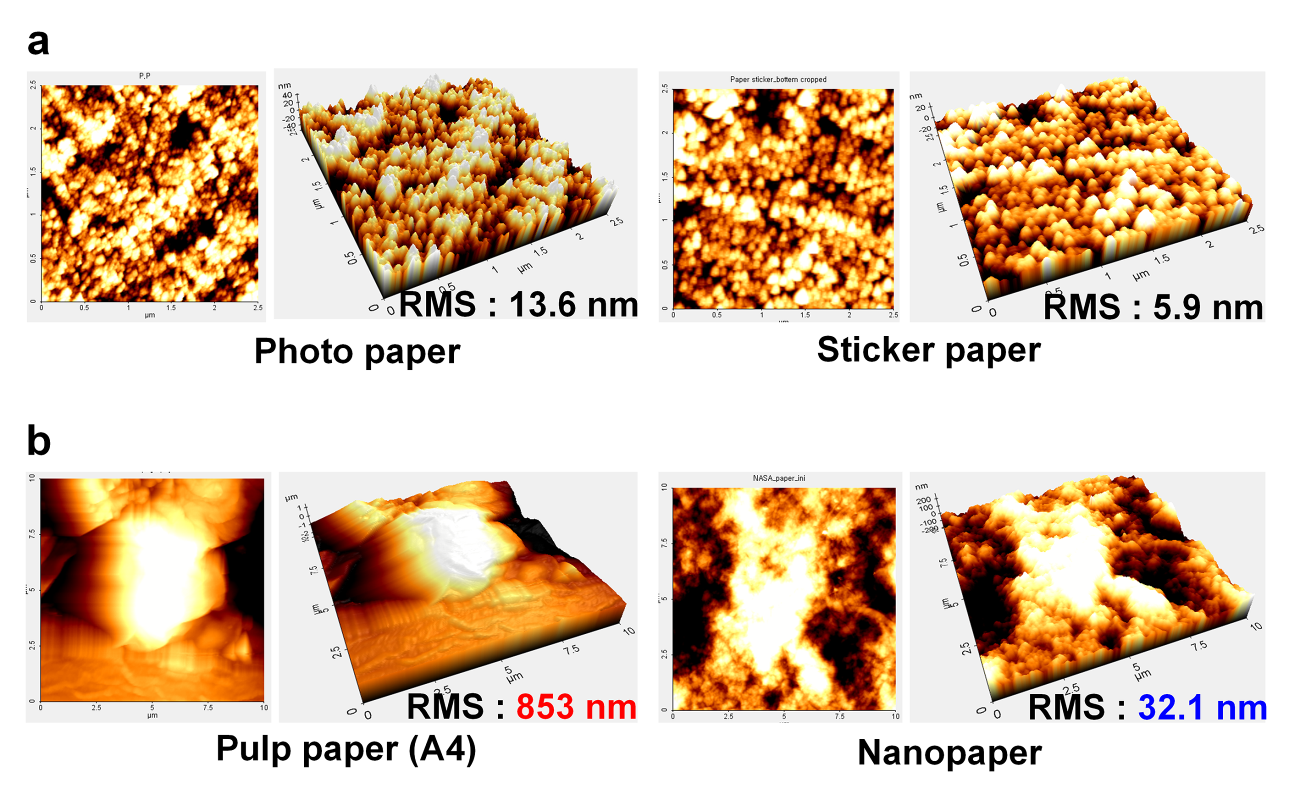


**Figure S5**. Analysis of surface morphology using AFM. (a) Results of papers with polymer coating on surface. (b) Results of pure wood-based papers without polymer coating.

**6. Characterization of pEGDMA**

Figure S6 shows the polymerization and characterization of pEGDMA. The polymerization process is provided in the manuscript. EGDMA was characterized by Fourier transform infrared spectroscopy (FT-IR), showing no difference between the EGDMA monomer and the polymerized pEGDMA. The results support the successful polymerization of the EGDMA monomer.


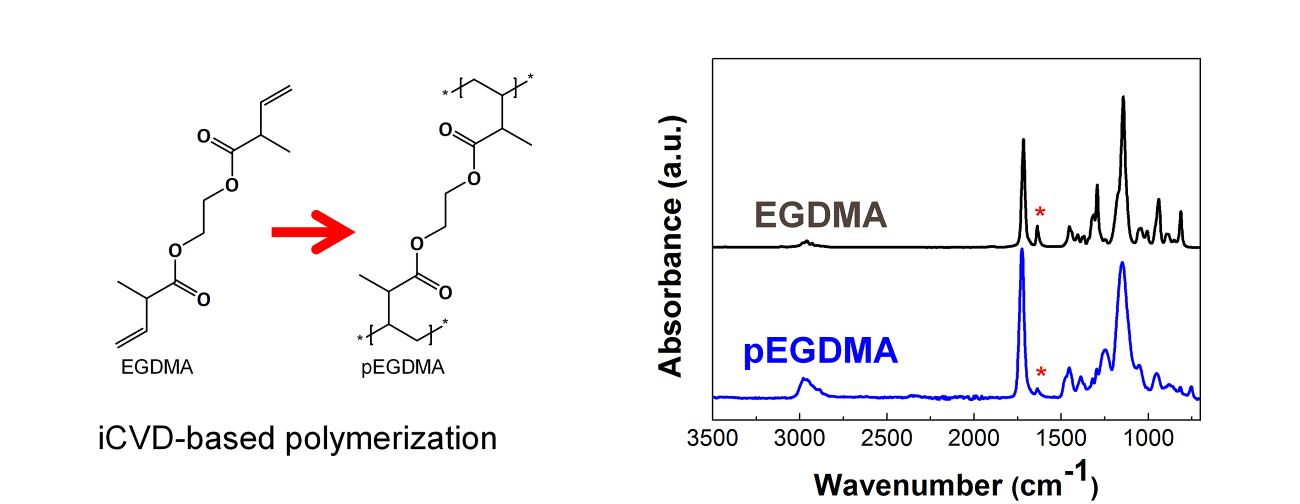


**Figure S6**. FT-IR spectra of EGDMA and pEGDMA. A decrease in the peak at 1750 cm^-1^ which corresponds to the vinyl moiety in the EGDMA monomer, is identically seen in the polymerized pEGDMA.

**REFERENCES**

1. Hua, P. & Ning, D. Electric characteristics and resistive switching mechanism of Ni/HfO_2_/Pt resistive random access memory cell. *Acta Phys. Sin-Ch. Ed.* 63, 147301 (2014)
2. Lee, B.–H. et al. Direct Observation of a Carbon Filament in Water-Resistant Organic Memory. ACS Nano. 9. 7306—7311 (2015)
3. Hsieh, M. –C., Kim, C., Nogi, M. & Suganuma, K. Electrically conductive lines on cellulose nanopaper for flexible electrical devices. *Nanoscale* 5, 9289—9295 (2013)
